# Evaluation of Mental Health and Well-Being Among Kansas Pharmacists Post-COVID-19 Pandemic

**DOI:** 10.7759/cureus.65962

**Published:** 2024-08-01

**Authors:** Saajan H Bhakta, Sainamitha R Palnati, Pooja Patel, Bradley J Newell

**Affiliations:** 1 Department of Research, Kansas College of Osteopathic Medicine, Wichita, USA; 2 Pharmacology, Dandurand Drug, Wichita, USA; 3 Department of Pharmacy Practice, University of Kansas School of Pharmacy – Wichita, Wichita, USA

**Keywords:** psychological well-being, psychological stressors, rural health, pharmacy, pharmacists, midwestern usa, mental health, kansas, covid-19 pandemic, burnout

## Abstract

Background

COVID-19 has profoundly affected pharmacists, causing burnout from heavier workloads, personal stressors, and disrupted healthcare delivery. Research on pharmacists' mental health during the pandemic, especially in rural areas like Kansas, remains limited.

Objectives

This study aimed to understand perceptions, experiences, and impacts on the mental, emotional, and psychological well-being of active Kansas pharmacists during the COVID-19 pandemic, including evaluating workplace modifications on mental health.

Methods

Kansas licensed pharmacists were recruited via email distributions through five Kansas pharmacy organizations and informal referrals among colleagues. After consenting, respondents completed a 15-minute, 28-question survey via Qualtrics. The survey included 11 questions concerning demographics and employment characteristics, along with 17 questions designed to assess the impact of COVID-19 on mental health, structured according to existing literature. Participation was uncompensated, and incomplete surveys were omitted from the analysis.

Results

One hundred and seven respondents (83.59% completion) represented 3.25% of Kansas's 3,290 pharmacists. They were aged 26-66 (M=38.7), the majority female (72.57%) and white (84.84%), with 14.24 years average practice duration (SD=10.94). Data covered 12 rural and 11 urban counties, with 50.91% staff pharmacists and 22.73% pharmacy managers. Many worked over 40 hours weekly in 13 settings. Findings showed increased workload (24.68%), medication shortages (24.03%), and burnout (24.32%) affecting job considerations. Workplace changes impacted personal mental health, with the main stressors being work-related factors (19.21%), social distancing (18.95%), and health concerns (12.63%).

Conclusion

This study underscores the pandemic's profound toll on Kansas pharmacists' mental, emotional, and physical health, leading to burnout, job dissatisfaction, and decreased effectiveness. It emphasizes the urgency of organizational interventions.

## Introduction

The COVID-19 pandemic has not only reshaped our daily lives but has also left lasting impacts on society, resulting in stressors that have placed an overwhelming burden on healthcare workers [1]. Factors such as increased workloads, inconsistent expectations from leadership, inadequate resources, labor shortages, and staffing challenges have led to heightened exhaustion and burnout among healthcare professionals [2,3]. Similarly, the pandemic affected social and personal factors such as family commitments, financial stressors, and social distancing, which have increased the risk of burnout. Individuals experienced fatigue more readily before and after a typical workday, which exposed them to a higher risk of burnout, mental health distress, and an overall lack of wellness [2-9]. The pandemic has significantly disrupted healthcare delivery, heightening stress levels for all healthcare professionals, including pharmacists [9,10]. This is evident in a notable surge in the risk and prevalence of burnout among pharmacists throughout the United States of America.

While recent studies have highlighted the challenges faced by healthcare workers such as nurses and physicians with frequent changes, there is a research gap concerning the mental health and well-being impacts of the pandemic on pharmacists, particularly in rural settings across states like Kansas [2,4,6,7,10]. Recognizing the nuances of burnout, emotional exhaustion, and compassion fatigue is crucial in providing support for this vital sector of the healthcare system. Research on burnout has adopted a grounded theory approach with a focus on the antecedents and consequences of burnout, emphasizing both individual- and organizational-focused interventions [5]. This approach can guide an understanding of how to mitigate stressors through healthcare policies that promote collaboration, stress management, and overall well-being, including work-life balance. To address the prevalence and consequences of burnout, emotional exhaustion, and compassion fatigue among pharmacists, despite high levels of personal accomplishment, efficacy, and compassion satisfaction, a qualitative survey was designed and administered to measure these variables among licensed pharmacists working in Kansas [3,5,10].

Objectives

The primary objective of this study was to understand the perceptions and lived experiences of the mental, emotional, and psychological health of practicing Kansas pharmacists during the COVID-19 pandemic. The secondary outcome attempts to understand the impact of workplace changes and adaptations on the personal mental health of practicing pharmacists in Kansas.

Previously presented

This work has not been previously published. It was presented as a poster presentation at the 2023 Kansas Association of Osteopathic Medicine Mid-Year CME Conference on November 10th, 2023, and at the 2023 Kansas College of Osteopathic Medicine Research Day on May 5th, 2023. Additionally, the work was presented in an invited symposium at the 2023 American Psychological Association Annual Convention in Washington, D.C., on August 2nd, 2023. This work is expected to be presented as a poster presentation at the Pharmacy Education 2024: American Association of College of Pharmacy annual meeting on July 21st-22nd, 2024, in Boston, MA. Also, this work is expected to be presented as an abstract in the American Journal of Pharmaceutical Education, August-September 2024 issue.

## Materials and methods

Study design

This study was a cross-sectional survey. Licensed pharmacists in Kansas were recruited online utilizing emails distributed through five Kansas pharmacy organizations (Kansas Pharmacists Association, Wichita Academy of Pharmacists, Kansas Health System Pharmacists, American Society of Consultant Pharmacists-Kansas Chapter, Currus Independent Pharmacies of Kansas), as well as via word-of-mouth amongst colleagues. Data was collected digitally online between February 25, 2023, and April 31, 2023. The inclusion criteria included the following: individuals must be between the ages of 18 and 85, have practiced as a licensed pharmacist in the state of Kansas during the COVID-19 pandemic, must be able to read and write in English, and must possess the technical capability to use a computer, tablet, or mobile device to complete a digital survey. In this study, the COVID-19 pandemic is defined as the time period of March 11, 2020, and thereafter, as the World Health Organization declared COVID-19 a pandemic on March 11, 2020 [1]. All inclusion criteria were determined based on self-reported data after review and the digital informed consent agreement. The exclusion criteria included individuals younger than the age of 18 or older than the age of 85, individuals who did not practice as a licensed pharmacist in the state of Kansas during the COVID-19 pandemic, individuals who cannot read and write in English, and individuals who do not possess the technical capability to use a computer, tablet, or mobile device to complete a digital survey. This study utilized a convenience sampling method, where participants were selected based on their accessibility through pharmacy organizations and word-of-mouth. Participants self-reported their eligibility based on predefined inclusion criteria. After obtaining informed consent, respondents were voluntarily asked to complete a 15-minute, 28-question survey about their mental health experiences during the pandemic (Table 1). The survey questionnaire, which was administered through Qualtrics, consisted of 11 questions on demographics and employment characteristics, followed by 17 questions assessing the impact of COVID-19 on mental health. The survey instrument was developed using existing literature [2-11] as a foundation. Although empirical testing or a pilot test was not conducted, the instrument was constructed based on established methods and prior research findings [2-11]. No incentive was offered for participation. Incomplete survey responses were excluded from the study. This study received exemption approval from two institutional review boards: the Kansas College of Osteopathic Medicine and the University of Kansas Medical Center in Wichita. The Kansas College of Osteopathic Medicine granted an exemption on February 1, 2023, and the University of Kansas Medical Center-Wichita provided its exemption on February 1, 2023.

**Table 1:** Survey questionnaire on impact of COVID-19 on mental health Q: question

Survey Question Number	Question
Q1-11	Demographic and employment questions
Q12	During the COVID-19 pandemic, were you:
	Single
	In a committed partnership of any kind
Q13	During the COVID-19 pandemic, did you identify as a primary caregiver for any other individual(s) or dependent(s)?
Q14	For each statement below, please select how much you agree or disagree with the statement:
	I always find new and interesting aspects in my work.
	There are days when I feel tired before I arrive to work.
	It happens more and more often I talk about work in a negative way.
	During the pandemic, after work, I needed more time than in the past to relax and feel better.
	I find my work to be a positive challenge.
	During the pandemic, I began to think less at work and do my job mechanically.
	During the pandemic, I became disconnected from my type of work.
	After work, I had enough energy for my leisure activities.
	After work, I am worn out and weary.
	When I work, I usually feel energized and engaged with my work.
	I can manage the amount of my work well.
Q15	How would you describe the pandemic’s impact on your mental well-being? (Select ALL that apply)
	My workload has increased during the pandemic.
	Although out of my control, I feel burdened by the increased medication shortages during the COVID-19 pandemic.
	I am concerned that my workplace exposure puts me at an increased risk of COVID-19.
	I feel more anxious since the start of the COVID-19 pandemic.
	I would like my company to provide mental health services specific for pharmacists.
	I am overwhelmed by patients’ frequent COVID-19 related questions.
	I have experienced an increased amount of harassment by patients since the start of the COVID-19 pandemic.
Q16	What, if any, do you think have been positive outcomes from the COVID-19 pandemic? (Select ALL that apply)
	More efficient processes in practice
	Improved working relationships and collaboration with peers and colleagues
	Enhanced opportunities for professional development
	Increased wages, incentives, and bonuses
	Increased personal appreciation and satisfaction of life and work
	Other: Please specify
	There have been no positive impacts of the COVID-19 pandemic
Q17	During the COVID-19 pandemic, did your employer or workplace provide sufficient mental and emotional health support and resources during the pandemic that you are aware of?
Q18	From the list below, and if any, what are the 3 things that you feel would support your wellbeing at work most significantly? (Select up to 3)
	Increased paid time away
	Enhanced wages and financial incentives
	Free onsite mental health counseling
	Access to free offsite mental health counseling
	Resilience and stress management workshops
	Designated lunch break with the pharmacy being closed for business and operation
	A remote working option
	Other: Please specify
Q19	During the COVID-19 pandemic, did you use paid time off, including sick days?
Q20	During the COVID-19 pandemic, did you experience a sense of guilt or shame when requesting paid time away?
Q21	Were there any changes to your work environment because of the COVID-19 pandemic? (Select ALL that apply)
	Scheduling changes
	Overtime work
	Increased administrative or technician-related duties
	Staffing and labor challenges
	Negative impact on quality patient care (medication errors, etc.)
	Precepting requirements
	Other: Please specify
Q22	Were you satisfied with your job or job role during the COVID-19 pandemic?
Q23	If you considered leaving your position or changing your job role during the pandemic, please select all of the reasons why.
	I did not consider leaving my position or job.
	Burnout (Feelings of energy depletion or exhaustion; Increased mental distance, or feelings of negativism or cynicism related to one's job; Reduced professional efficacy; World Health Organization, 2011).
	Workplace policies
	PPE requirements
	Inadequate resources (labor, time, inventory)
	Inconsistent expectations from leadership
	Wage and financial reasons
	Other reasons: Please specify
Q24	Did your job give you flexibility to meet the needs of both your work and personal life, during the COVID-19 pandemic?
Q25	During the COVID-19 pandemic, do you believe you had a healthy work-life balance?
Q26	During the COVID 19 pandemic, what were the stressors impacting your personal well-being. Please select all that apply.
	Family commitments
	Financial stressors
	Housing
	Health (physical, emotional, and mental)
	Caregiver obligations
	Work
	Political activities
	Social distancing and the lack of social interaction
	The fear of contracting COVID-19
	Professional uncertainty
Q27	Were you under the care of a licensed professional for mental health support prior to March 10, 2020?
Q28	During the COVID-19 pandemic, did you receive mental health care or counseling?

Population

As of 2022, there were 3,290 licensed pharmacists in the state of Kansas [11]. Respondent data was included for all pharmacists 18 years of age or older practicing in Kansas during the COVID-19 pandemic. Licensed pharmacists in any practice setting were included throughout the 105 counties of Kansas. Survey responses were collected via Qualtrics from February 2023 to April 2023. The completion of the survey was voluntary and anonymous. A total of 107 respondents completed the survey out of 125 attempted surveys (83.59% completion rate).

Outcomes

This study aimed to explore the psychological well-being of pharmacists practicing in Kansas amid the COVID-19 pandemic. Moreover, recommendations were sought out from practicing pharmacists and pharmacy leaders on strategies for sustainable mental health interventions to support this group of healthcare professionals. The overarching goal was to provide insightful recommendations to employers and policymakers, facilitating meaningful and effective changes in support of the mental health of these professionals.

Statistical analysis

The statistical analyses conducted in this study were designed to comprehensively evaluate the impact of COVID-19 on the mental health and work-life balance of Kansas pharmacists. Baseline characteristics were analyzed using descriptive statistics, which included gender, age, sexual orientation, and race/ethnicity. Respondents’ ages were condensed to age ranges for descriptive statistics. Age ranges were categorized into seven groups: <20, 21-30, 31-40, 41-50, 51-60, 61-70, and >71 years old (Table 2).

**Table 2:** Demographics and employment characteristics n: number of respondents ^a^Not all respondents answered all questions.

Characteristic	Respondents	Confidence Interval (95%)
Age Groups, n (%)	n = 88^a^	2.25
21-30 years old	20 (22.73%)	
31-40 years old	40 (45.45%)	
41-50 years old	12 (13.64%)	
51-60 years old	12 (13.64%)	
61-70 years old	4 (4.55%)	
> 71 years old	0 (0.00%)	
Gender, n (%)	n = 103^a^	91.69%
Male	29 (28.16%)	
Female	74 (71.84%)	
Other	0 (0.00%)	
Prefer not to disclose	1 (0.97%)	
Race/Ethnicity, n (%)	n = 104^a^	30.59%
Hispanic/Latino	1 (0.96%)	
American Indian or Alaska Native	0 (0.00%)	
Asian	7 (6.73%)	
Middle Eastern	2 (1.92%)	
Black or African American	1 (0.96%)	
Pacific Islander	0 (0.00%)	
White	90 (86.54%)	
European American	3 (2.88%)	
Unknown	0 (0.00%)	
Other: Please Specify	2 (1.92%)	
Sexual Orientation, n (%)	n = 103^a^	53.73%
Heterosexual	98 (95.15%)	
Homosexual	2 (1.94%)	
Bisexual	1 (0.97%)	
Pansexual	0 (0.00%)	
Asexual	1 (0.97%)	
Prefer not to disclose	1 (0.97%)	
Practice Setting, n (%)	n = 103^a^	7.36%
Ambulatory Care	5 (4.85%)	
Community-Based	34 (33.01%)	
Independent	12 (11.65%)	
Chain	22 (21.36%)	
Institutional	34 (33.01%)	
Managed-Care	0 (0.00%)	
Long-Term Care/Home Health/Hospice	1 (0.97%)	
Academia	3 (2.91%)	
Industry/Sales	0 (0.00%)	
Research & Development	0 (0.00%)	
Mail Order	1 (0.97%)	
Government (VA, FDA, Military)	2 (1.94%)	
Compounding/Infusion	1 (0.97%)	
Worked in multiple settings	18 (17.48%)	
Other	4 (3.88%)	
Pharmacist Role, n (%)	n = 100^a^	16.13%
Contract or Per Diem Staff Pharmacist	3 (3.00%)	
Float or Relief Staff Pharmacist	3 (3.00%)	
Staff Pharmacist	50 (50.00%)	
Pharmacy Manager	23 (23.00%)	
Director of Pharmacy	5 (5.00%)	
Other Executive Leader	3 (3.00%)	
Other	13 (13.00%)	
Weekly work hours, n (%)	n = 103^a^	37.55%
0-20 hours	8 (7.77%)	
21-35 hours	7 (6.80%)	
36-40 hours	31 (30.10%)	
40+ hours	57 (55.34%)	
Highest level of training, n (%)	n = 55^a^	38.59%
PGY1	31 (56.36%)	
PGY2	23 (41.81%)	
Fellowship	1 (1.81%)	
Board certification, n (%)	n = 50^a^	4.67%
AAHIVP	1 (2.00%)	
BCACP	10 (20.00%)	
BCCCP	2 (4.00%)	
BCGP	3 (6.00%)	
BCIDP	2 (4.00%)	
BCOP	3 (6.00%)	
BCPP	5 (10.00%)	
BCPPS	1 (2.00%)	
BCPS	22 (44.00%)	
CSP	1 (2.00%)	
Years practice experience, n (%)	n = 101^a^	2.16%
<1	0 (0.00%)	
1-5	23 (22.77%)	
6-10	30 (29.70%)	
11-15	14 (13.86%)	
16-20	8 (7.92%)	
21-25	8 (7.92%)	
26-30	3 (2.97%)	
31-35	10 (9.90%)	
36-40	3 (2.97%)	
41-55	2 (1.98%)	
56+	0 (0.00%)	
Kansas county or counties of practice, n (%)	n = 100^ a^	3.64%
Barton	1 (1.00%)	
Butler	1 (1.00%)	
Dickinson	1 (1.00%)	
Douglas	8 (8.00%)	
Ellis	4(4.00%)	
Finney	1 (1.00%)	
Geary	2 (2.00%)	
Harvey	1 (1.00%)	
Jackson	1 (1.00%)	
Johnson	29 (29.00%)	
Kearny	1(1.00%)	
Leavenworth	8 (8.00%)	
Lyon	2 (2.00%)	
Marshall	2 (2.00%)	
McPherson	1 (1.00%)	
Pratt	1 (1.00%)	
Reno	2 (2.00%)	
Riley	3 (3.00%)	
Saline	10 (10.00%)	
Sedgwick	20 (20.00%)	
Shawnee	5 (5.00%)	
Thomas	1 (1.00%)	
Wyandotte	28 (28.00%)	

To compare differences in perceived work-life balance across various demographic and categorical factors, independent sample t-tests and one-way ANOVAs were employed. These analyses were crucial for assessing whether gender, county classification (rural or urban), age, and relationship status had statistically significant effects on work-life balance. The use of these tests allowed for the examination of group differences and helped identify specific areas where interventions may be needed. Further, a one-sample chi-squared test was conducted using SPSS to evaluate whether there was a significant difference in the impact of COVID-19-related factors between male and female pharmacists. This test provided insights into potential gender disparities in how COVID-19 affected pharmacists, thus contributing to a deeper understanding of the pandemic's differential impact. To explore the interaction between multiple factors, a two-way contingency table analysis was performed. This analysis facilitated the investigation of the relationships between gender, county classification, and relationship status on perceived work-life balance and feelings of shame or guilt related to requesting paid time off. The post-hoc analysis was particularly important for refining these findings, allowing for a more nuanced understanding of how these factors intersect and influence each other.

Statistical analysis analyzed with Microsoft Excel and IBM Corp. Released 2021. IBM SPSS Statistics for Windows, Version 28.0. Armonk, NY: IBM Corp. was measured with an overall level of 0.05 for the primary and secondary outcome analyses. Independent sample t-tests and one-way ANOVAs were performed to compare differences between gender, county classification (rural or urban), age, and relationship status in having a healthy work-life balance. SPSS was used to perform a one-sample chi-squared test and two-way contingency table analysis using crosstabs. A one-sample chi-square test was conducted to determine whether women or men were more greatly affected by COVID-19-related factors.

Furthermore, a post-hoc analysis was made to further assess gender, classification of county of practice (rural or urban), and relationship status on perceived healthy work-life balance and experiences of feelings of shame or guilt in requesting paid time away.

## Results

Baseline characteristics

Demographics

A total of 107 respondents completed the voluntary survey (83.59% completion rate) from February 25 to April 31, 2023, which represented 3.25% of the total population of 3,290 practicing Kansas pharmacists [12]. Respondents were between ages 26 and 66 (M = 38.7). A total of 72.57% of respondents identified as female, and 84.84% identified as white. The majority of respondents identified as heterosexual (94.69%). The mean years of practice as a licensed pharmacist were 14.24 (SD = 10.94, range 1.5 to 42 years). There were 12 rural and 11 urban Kansas counties represented by the survey’s respondents. A majority of respondents reported practicing as a staff pharmacist (50.91%) or pharmacy manager (22.73%). Many pharmacists were working over 40 hours per week. Respondents represented over 13 practice settings. During the pandemic, 91.48% of respondents were in a committed partnership of any kind, and 46.15% identified as primary caregivers for other individuals and/or dependents. Table 2 summarizes the demographic and employment characteristics of the respondents.

Work-Life Balance and Job Satisfaction

A lack of a healthy work-life balance was indicated by 53 respondents (51.46%) due to work overload, inadequate resources, inconsistent leadership expectations, financial struggles, and burnout. A positive level of job satisfaction was expressed by 59 respondents (56.73%). There were 40 respondents (18.02%), who reported that they did not consider leaving their positions or changing jobs during the pandemic. There was a higher frequency of Kansas pharmacists practicing in urban counties agreeing with having a healthy work-life balance than compared to Kansas pharmacists from rural counties (p = 0.689).

Primary outcome

The primary outcome underscored the mental health impact of COVID-19, evident in burnout, job transitions, and active pursuit of resources. The findings revealed significant effects on Kansas pharmacists, notably heightened occurrences related to increased workload (24.68%) and medication shortages (24.03%), which were beyond their control. Burnout emerged as the foremost reported factor (24.32%) influencing the consideration of leaving their current position or transitioning to a different job amidst the pandemic. A minority of respondents (18.02%) indicated no contemplation of such position changes. Before March 10, 2020, a limited subset (11.54%) sought mental health support from licensed professionals. However, a notable increase (25.69%) was observed during the pandemic in respondents seeking mental health care and counseling.

Secondary outcome

**Figure 1:**
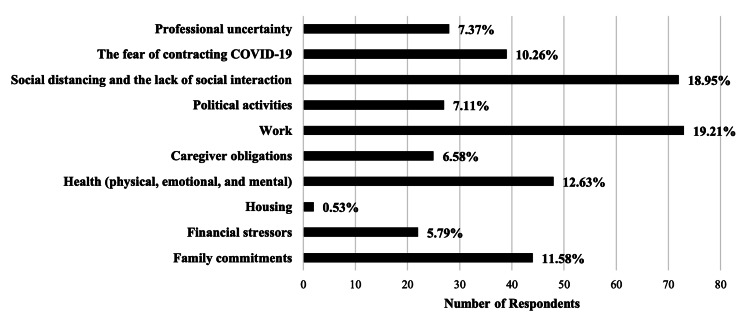
The highest reported stressors impacting the well-being of practicing Kansas pharmacists during the pandemic Confidence interval: 15.92

The secondary outcome of this study explores the effects of workplace changes and adaptations on the personal mental health of Kansas pharmacists during the COVID-19 pandemic. The analysis revealed that the predominant stressors impacting pharmacists were work-related issues (19.21%), challenges stemming from social distancing and diminished social interaction (18.95%), and concerns regarding physical, emotional, and mental health (12.63%) (Figure 1). These findings underscore the multifaceted nature of stress experienced by pharmacists, encompassing both direct workplace pressures and broader social factors.

Workplace Factors

**Figure 2:**
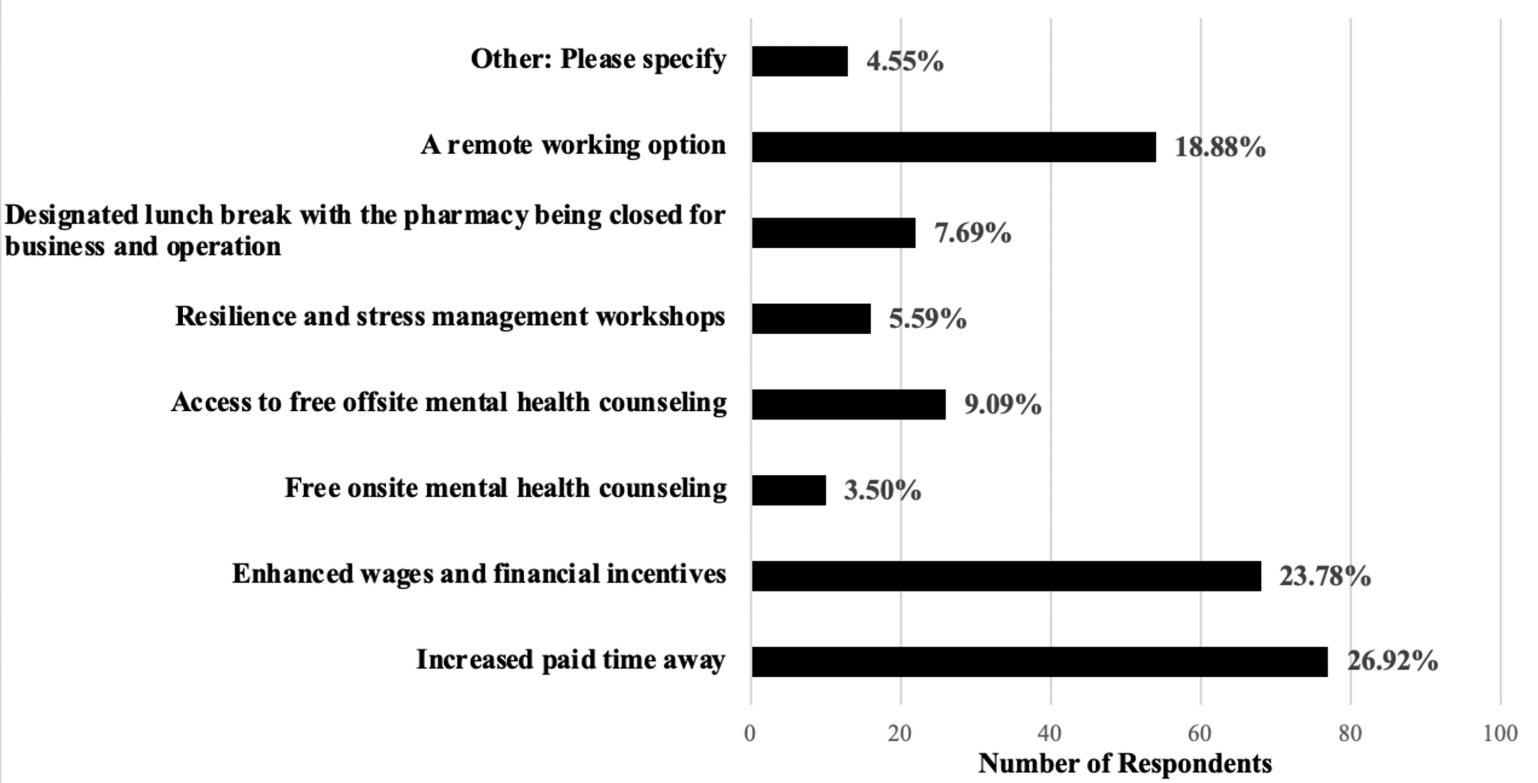
The highest reported recommendations from practicing pharmacists to Kansas employers and policymakers Other: please specify (4.55%), adequate staffing, improved work relationships, onsite childcare, streamlined communication with leadership, and team-focused decision-making

In terms of support needs, respondents indicated that enhanced support from employers could be achieved through increased paid time off (26.92%), improved wages and financial incentives (23.78%), and the provision of remote work options (18.88%) (Figure 2). These suggestions reflect a demand for greater flexibility and financial security, which are critical to alleviating the stress and burnout reported by participants. Despite these challenges, some positive outcomes were identified. Respondents noted that the pandemic led to more efficient processes in their practice (15.29%) and increased opportunities for professional development (17.83%). However, a significant portion (21.02%) felt that the pandemic had no positive impact on their professional lives.

Mental Health Support at the Workplace

Also, the study highlighted that 43.27% of respondents disagreed with the statement that their employers provided adequate mental health support during the pandemic. This dissatisfaction indicates a significant gap in the support structures available to pharmacists during this crisis. The pandemic's influence on mental well-being was notably associated with increased workload due to pharmacy staffing shortages (28.11%), heightened administrative responsibilities (16.37%), and changes in scheduling practices (18.86%). These factors, coupled with instances of harassment from patients and family members and challenges related to medication shortages, contributed to the overall mental health burden experienced by pharmacists. The comprehensive nature of these findings illustrates the complex interplay between workplace adaptations and personal mental health, highlighting the need for targeted interventions to support pharmacists effectively in future crises.

Post-hoc analysis

The post-hoc analysis further assessed feelings of guilt or shame when requesting paid time away and perceived experiences of a healthy work-life balance. Both variables were evaluated for differences within self-identified gender, classification of county of practice (rural or urban country in Kansas), and relationship status (single or in a committed partnership of any kind).

Feelings of Guilt or Shame when Requesting Paid Time Away

To assess whether practicing pharmacists experienced feelings of guilt or shame when requesting paid time away, a one-sample chi-square test was conducted. The results were not statistically significant, χ2 (2, N = 103) = 0.01, p = 0.922, as the categories of feeling guilt or shame and not feeling guilt or shame occurred with equal probabilities. The proportion of pharmacists who did not experience feelings of guilt or shame was equal to the hypothesized proportion of 0.50.

Furthermore, a two-way contingency analysis was conducted to evaluate whether females or males were experiencing more feelings of shame or guilt when requesting paid time away. The two variables were gender (male and female) and feelings when requesting paid time away (not feeling shame or guilt and feeling shame or guilt). Gender and experiences of feeling shame or guilt were not found to be significantly related; Pearson χ2 (4, N = 2) = 1.78, p = 0.339, Cramér’s V = 0.13. The proportions of respondents who experienced feelings of guilt or shame and self-identified as male or female were 0.31 and 0.69, respectively.

Healthy Work-Life Balance

For all respondents, a one-sample chi-square test was conducted to assess how practicing pharmacists felt about their healthy work-life balance. The results were significant, χ2 (4, N = 101) = 16.97, p = 0.002 for ‘definitely disagree’ that they had a healthy work-life balance. The proportion of pharmacists who did not experience a healthy work-life balance was greater than the combined hypothesized proportion of 0.40 for respondents to select disagree or strongly disagree. The results were significant: χ2 (4, N = 101) = 15.68, p = 0.003.

Also, differences between self-identified genders in having a healthy work-life balance were further analyzed. A two-way contingency analysis was conducted to evaluate whether more females or males believed they had a healthy work-life balance. The two variables were gender (male and female) and perceived work-life balance. Gender and perceived work-life balance were not found to be statistically significant; Pearson χ2 (12, N = 101) = 11.70, p = 0.585, Cramér’s V = 0.20 (Table 3).

**Table 3:** Changes in healthy work-life balance based on respondent characteristics during COVID-19

Respondent Characteristic	Pearson chi-square	p-value (alpha)	Cramer’s V
Gender (Male, Female, or Prefer not to disclose)	11.70	0.585	0.20
Relationship Status (Single or in a committed partnership of any kind)	3.54	0.313	0.19
County Classification (Rural or Urban)	1.41	0.872	0.12

Similarly, a two-way contingency analysis was conducted to evaluate whether more practicing pharmacists in urban or rural counties in Kansas believed they had a healthy work-life balance. The two variables were county (rural and urban) and perceived work-life balance. County classification and perceived work-life balance were not found to be statistically significant; Pearson χ2 (4, N = 101) = 3.54, p = 0.313, Cramér’s V = 0.19 (Table 3).

Additionally, a two-way contingency analysis was conducted to evaluate whether individuals who shared their relationship status (single and in a committed relationship of any kind) believed they had a healthy work-life balance. The two variables were relationship status (single and in a committed relationship of any kind) and perceived work-life balance. Relationship status and perceived work-life balance were not found to be statistically significant; Pearson χ2 (4, N = 95) = 1.41, p = 0.872, Cramér’s V = 0.12 (Table 3).

## Discussion

To the best of this research team’s knowledge, this is the first study exploring the mental, emotional, and psychological impacts of the COVID-19 pandemic on pharmacists in Kansas. This study aimed to understand the support needs and make recommendations to leaders on ways to improve the quality of well-being for pharmacists in Kansas. As hypothesized, a significant proportion of respondents acknowledged the absence of a healthy work-life balance and experienced adverse effects due to COVID-19. Overall, we identified 51.46% of pharmacists who shared that they lacked a healthy work-life balance due to various occupational and personal stressors, which impacted their well-being.

Job satisfaction

Contrarily, our findings revealed that the majority also expressed an overall positive level of job satisfaction. Similar results were obtained in another study, which reported that burnout was higher among pharmacists who did not enjoy work (96%) than compared to those who did enjoy work (62.4%) [6]. The study suggested that personal satisfaction and accomplishment outweigh the increased risk of burnout during stressful, challenging times such as the pandemic [6].

Additionally, our study indicated a minority of participants contemplating leaving their current position or pursuing a job change during the pandemic, and other studies have attributed similar results to high levels of burnout [11]. Comparable results have been reported in other healthcare professionals (physicians, nurses, etc.) and employees of departments at academic hospitals (case management, pharmacy, clinical trials, clinical nutrition, etc.), as 70% of healthcare employees expressed additional personal stressors outside of work due to the pandemic [4]. A recent study suggested that lower levels of occupational stress were coupled with higher job performance and increased psychological well-being [11]. Furthermore, a previous study shared the possibility of job satisfaction increasing with age and experience, which reduces exhaustion and disengagement at the workplace [6].

Recommendations from pharmacists

The present study had many recommendations suggested by respondents to employers and lawmakers to improve work-life balance and well-being. In this study, respondents reported seeking increased paid time off (26.92%) and lunch breaks (7.69%), which may be granted through allowing more paid time off for pharmacists to attend healthcare visits that can be reinforced via required proof of visits. Similarly, a recent study reported that a majority of pharmacists had not taken any time off work for sick leave, and many hoped to take time off work but felt as if they were unable to do so during the pandemic [6].

Mental health support

Additionally, a significant portion of respondents in our study reported a desire for more available options and/or time for therapy (43.27%), as they believed their employer or workplace did not provide sufficient mental and emotional health support and resources during the pandemic that they were aware of. A proposal for more flexible times of availability for therapy would be to offer more telehealth hours outside of traditional business hours. Therapists supporting the healthcare community can consider offering non-standard therapy hours, such as early mornings or late evenings. As a result of our data, we recommend therapists consider traveling or offering rural therapy days by making efforts to reach those needing support who choose not to use or are averse to telemental health. This can ensure that such individuals may still have access to care in non-urban settings.

Unfortunately, pharmacists reported being overwhelmed by patients' frequent COVID-19 questions (5.84%), increased patient harassment (10.39%), and political activities (7.11%). These results yield a recommendation to provide pharmacists with specific types of counselors and offices to direct their patients towards, which can be especially beneficial for patients who have increased disease states or mental health effects correlated to their pharmacy care. Nonetheless, it is critical to spread awareness and education for your practice specialty as a psychologist, therapist, counselor, etc., within your local community through visits to pharmacies to share information on the types of support and care you provide (psycho-oncology, health psychology, palliative and hospice psychology, etc.).

Interprofessional collaboration

Interprofessional collaboration is needed to provide sustainable, effective individual and organization-focused interventions that reinforce well-being policies of support and care. Researchers have discovered that many employers' current practices for stress management are incompatible with providing quality mental health and well-being services to employees [6]. This can be addressed by fostering a supportive work environment that values and prioritizes mental health, which can be achieved through several initiatives. A culture of open communication will encourage pharmacists and other healthcare professionals to express their feelings and concerns without fear of judgment or reprisal [13]. This enables team members to not only share their experiences but also seek support from colleagues. The opportunities of regular debriefing sessions can create safe spaces where healthcare professionals can debrief and discuss challenging cases or experiences. These sessions can help them process their emotions and seek guidance from peers or supervisors [13]. Additionally, the implementation of established peer support networks or mentorship programs can provide a platform for experienced professionals to offer guidance and support to those experiencing burnout or emotional exhaustion. This can help create a sense of community and reduce feelings of isolation. Moreover, offering mental health resources and training at the workplace will strengthen a supportive work environment and community [14]. Organizational interventions yielded greater effectiveness compared to individual-focused interventions [14]. Some examples of organizational-focused strategies include improvements in work-life integration (altering scheduling and leave policies), changes in workload and workflow, and positive changes to the organization's values and culture [5]. A study among physicians had shown that organizational-focused interventions had a two-fold greater reduction in burnout than individual-focused interventions [5]. Though it is unknown if similar interventions are effective in reducing burnout among pharmacists, pharmacists face similar daily stressors at the workplace as physicians [5]. Individual strategies served a limited role in reducing burnout because they did not address the causes and contributors of burnout as compared to organizational strategies [3]. However, employing both strategies is likely essential for addressing burnout effectively [14]. Mental health awareness and resilience training consist of workshops or training sessions on topics such as stress management, resilience, and self-care [3]. These programs can equip healthcare professionals with practical skills and strategies to cope with work-related stressors and build psychological resilience.

The current literature is inconclusive regarding the most effective ways to enhance an individual's resilience and measure its impact on other team members [15]. Despite its popularity, resiliency training fails to address the issue of preventable occupational suffering caused by systemic problems that can be mitigated through organizational changes [16]. Imposing mandatory mindfulness training can lead to resentment and disengagement. Offering individual-focused mindfulness interventions or other choices among a range of options is a reasonable approach [13,16]. Some individual interventions are integrated into organizational initiatives aimed at supporting clinicians. An example of this is Schwartz Center Rounds [17]. These rounds provide clinicians with regular opportunities to discuss the social and emotional challenges they encounter while caring for patients and their families. Alongside this, introducing regular mental health screening and assessments can help identify early signs of burnout or emotional distress in individuals, which enables employers to offer additional support or interventions. On a national level, employee assistance programs (EAPs) can offer confidential counseling services, both in-person and online, to provide immediate support for mental health concerns [17]. EAPs can connect healthcare professionals to licensed therapists or counselors nationwide who specialize in stress management, burnout, and emotional well-being. The Resource Compendium for Health Worker Well-Being, provided by the National Academy of Medicine, is a compilation of measurement tools that are grounded in evidence-based practices [17]. These tools can be utilized by organizations to assess the well-being of their workforce. 

Undoubtedly, healthcare professionals should begin to prioritize their own well-being through self-care practices and set boundaries between work and personal life. This can include granting time for relaxation, regular exercise, healthy eating, adequate sleep, and engaging in hobbies and social connections. Furthermore, self-care should involve promoting psychological well-being, such as engaging in mindfulness or stress reduction techniques and seeking support from friends, family, or professional counselors.

Limitations

This study, while offering valuable insights into the impact of COVID-19 on pharmacists in Kansas, has several limitations that may influence the generalizability and interpretation of the findings. First, recall bias is a concern, as participants were required to reflect on their mental state and professional practices during the pandemic. This reliance on retrospective reporting may have introduced inaccuracies in the participants' recollections of their experiences and stressors. Second, the recruitment strategy, which relied on email distributions through pharmacy organizations and informal referrals, may have led to sampling and recruitment biases. The voluntary nature of participation, coupled with limited accessibility via email and the lack of widespread awareness campaigns, might have affected the representativeness of the sample. Moreover, the study's use of an online survey could have introduced sampling bias, excluding individuals without reliable internet access or those less comfortable with digital tools. The demographic composition of the sample, with a predominance of female (72.57%) and white (84.84%) respondents, may not fully reflect the diversity of the pharmacist population in Kansas, potentially impacting the generalizability of the findings. Additionally, the survey's structured nature, which did not include open-ended questions, might have restricted the depth and variety of responses, limiting the ability to capture the full range of participants' experiences and perceptions. Despite developing the survey based on established literature and pre-existing surveys, the absence of a pilot test means that the reliability and validity of the instrument have not been empirically verified. Consequently, there is a risk that the survey may not accurately or consistently measure the intended constructs, potentially affecting the precision and dependability of the collected data across various respondents. Lastly, the exclusion of incomplete surveys from the analysis, despite a high completion rate (83.59%), could introduce bias if incomplete responses differ systematically from complete ones, potentially affecting the robustness of the findings. Overall, while this study sheds light on the mental health impacts of the COVID-19 pandemic on Kansas pharmacists, these limitations should be considered, and future research could benefit from addressing these biases, incorporating more diverse and qualitative methods, and ensuring thorough testing of survey instruments to enhance the comprehensiveness and accuracy of the findings.

## Conclusions

This study supports the effect of the pandemic on the mental, emotional, and physical health of Kansas pharmacists. This resulted in burnout, feelings of negativity related to the job, and reduced professional efficacy. Burnout is a problem rooted in the workplace that necessitates solutions at the organizational level, focusing on systemic and systems-oriented approaches. Thus, this study advocates a call to action among employers and policymakers to address concerns and implement effective support strategies for pharmacists experiencing burnout, compassion fatigue, and emotional exhaustion.
